# Integrated transcriptome, small RNA and degradome sequencing approaches proffer insights into chlorogenic acid biosynthesis in leafy sweet potato

**DOI:** 10.1371/journal.pone.0245266

**Published:** 2021-01-22

**Authors:** Yi Liu, Wenjin Su, Lianjun Wang, Jian Lei, Shasha Chai, Wenying Zhang, Xinsun Yang

**Affiliations:** 1 Engineering Research Centre of Ecology and Agricultural Use of Wetland, Ministry of Education/Hubei Collaborative Innovation Center for Grain Industry, Yangtze University, Jingzhou, China; 2 Food Crops Institute, Hubei Academy of Agricultural Sciences/Hubei Engineering and Technology Research Centre of Sweet Potato/Hubei Key Laboratory of Food Crop Germplasm and Genetic Improvement, Wuhan, China; East Carolina University, UNITED STATES

## Abstract

Leafy sweet potato is rich in total phenolics (TP) which play key roles in health protection, the chlorogenic acid (CGA) constitutes the major components of phenolic compounds in leafy sweet potato. Unfortunately, the mechanism of CGA biosynthesis in leafy sweet potato is unclear. To dissect the mechanisms of CGA biosynthesis, we performed transcriptome, small RNA (sRNA) and degradome sequencing of one low-CGA content and one high-CGA content genotype at two stages. A total of 2,333 common differentially expressed genes (DEGs) were identified, and the enriched DEGs were related to photosynthesis, starch and sucrose metabolism and phenylpropanoid biosynthesis. The functional genes, such as CCR, CCoAOMT and HCT in the CGA biosynthetic pathway were down-regulated, indicating that the way to lignin was altered, and two possible CGA biosynthetic routes were hypothesized. A total of 38 DE miRNAs were identified, and 1,799 targets were predicated for 38 DE miRNAs by using in silico approaches. The target genes were enriched in lignin and phenylpropanoid catabolic processes. Transcription factors (TFs) such as apetala2/ethylene response factor (AP2/ERF) and Squamosa promoter binding protein-like (SPL) predicated in silico were validated by degradome sequencing. Association analysis of the DE miRNAs and transcriptome datasets identified that miR156 family negatively targeted AP2/ERF and SPL. Six mRNAs and six miRNAs were validated by qRT-PCR, and the results showed that the expression levels of the mRNAs and miRNAs were consistent with the sequencing data. This study established comprehensive functional genomic resources for the CGA biosynthesis, and provided insights into the molecular mechanisms involving in this process. The results also enabled the first perceptions of the regulatory roles of mRNAs and miRNAs, and offered candidate genes for leafy sweet potato improvements.

## Introduction

Sweet potato (*Ipomoea batatas* (L.) Lam.) is the seventh most important food crop in the world due to its wide adaptability, high nutrition and high productivity [1]. In the past, the tuberous roots of sweet potato were the main harvested organs. However, in recent years, the tender stems, and leaves of certain sweet potato varieties consuming as fresh vegetables is popular in many regions [2]. In central and southern China, leafy sweet potato contributes enormous economic values to the farmers. Its yield exceeds 75,000 kg/ha each year with the price about 0.59 USD/kg, making the total output value reach as much as 44,117 USD/ha [3]. Thus, planting leafy sweet potato is a commercially viable venture.

**Fig 1:**
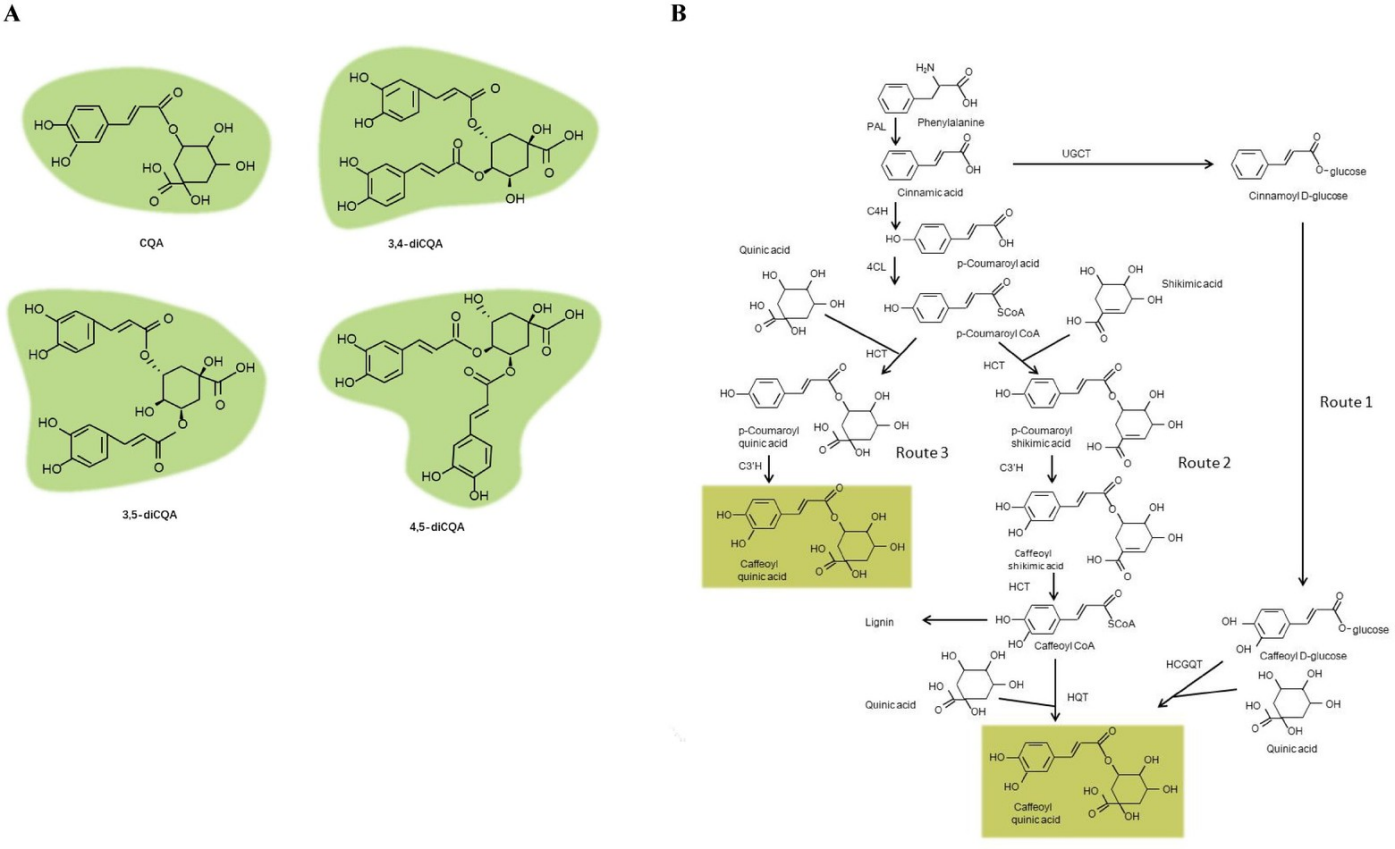
Specialized CGAs found in leafy sweet potato. (A) Structures of major CGAs. (B) The proposed pathway responsible for the CGA biosynthesis [18].

The nutritional attributes of leafy sweet potato are increasingly being recognized. It is rich in vitamins, minerals, dietary fibres, phenolics and proteins. These characteristics make it a candidate vegetable for reducing malnutrition [4,5]. Among these nutritional components, phenolics have attracted particular attention because they can reduce the risks of serious human afflictions, such as cancer and cardiovascular diseases, and protect the human body from oxidative stress which causes fatigue and aging [6–9]. Phenolics are the most abundant type of secondary metabolites produced in leafy sweet potato [10]. The major phenolic components in leafy sweet potato are caffeoylquinic acid derivatives, including caffeoylquinic acid (CQA), 3,4-O-dicaffeoylquinic acid (3,4-diCQA), 3,5-O-dicaffeoylquinic acid (3,5-diCQA) and 4,5-O-dicaffeoylquinic acid (4,5-diCQA) which belong to the CGA family [11–13] (Fig 1A).

Currently, the steps in the phenylpropanoid metabolic pathway that lead to CGA biosynthesis has been extensively reviewed in plants [14–18]. An overview of this pathway was summarized in Fig 1B: In route 1, hydroxycinnamoyl D-glucose: quinate hydroxycinnamoyl transferase (HCGQT) catalysed the formation of CGAs from caffeoyl D-glucoside and quinic acid in the roots of sweet potato [19,20]. In route 2, enzyme hydroxycinnamoyl-CoA quinate hydroxycinnamoyl transferase (HQT) catalysed the formation of CGAs from caffeoyl-CoA and quinic acid in tomato [21,22]. In route 3, p-coumaroyl quinate is synthesized by the catalysis of hydroxycinnamoyl-CoA (HCT), and subsequently hydroxylated by p-coumarate 3′-hydroxylase (C3′H) to form CGAs in *Arabidopsis* [23,24].

Gene studies showed that functional genes and TFs in the pathway were closely related to the biosynthesis of CGAs. A transesterification reaction between caffeoyl D-glucose and D-quinc acid was discovered in the CGA biosynthetic pathway of the roots of sweet potato via the isotope tracer method [20]. In addition, HCGQT extracted from sweet potato roots was found to catalyse the formation of CGAs in *in vitro* experiments [19]. It has been noted that high level of *HCT* expression could increase CGA accumulation in *Solanaceous* species [25]. In *Lonicera japonica*, the *HQT* gene was found to positively regulate CGA biosynthesis [26]. Overexpression of *HQT* gene isolated from *Cynara cardunculus* var. *scolymus* in *Nicotiana tabacum* led to rechannel of the phenylpropanoid pathway [27]. Some TFs were also reported to regulate the biosynthesis of CGA. *MYB1* was an important transcriptional activator of *PAL1*, while *MYB* 3 and 5 were found to act on the promotor of *PAL3* in carrots [28]. The biosynthesis of many phenolic compounds was also recorded to be regulated by the *WRKY* family, for example, *WRKYs* 38, 45, 60, 89 and 93 acted as activators for *HCT2* in poplar [29]. In *Salvia miltiorrhiza*, *AP2/EFR1* was reported to be able to increase the phenolic acid level [30]. However, despite the abundance of CGA compounds in sweet potato leaves, which is far more than that of the roots [29], and growing recognition of their importance to human health, there were few data in the literatures concerning genes involved in the CGA biosynthetic pathway in leafy sweet potato.

In addition, small non-coding RNAs had been extensively studied to be participated in epigenetic regulations by altering gene expression. Small RNAs, such as miRNAs, composed a class of endogenous small non-coding RNAs that ranged from approximately 20 to 24 nt in length. The small RNA negatively regulated the expression of their target genes at the post-transcriptional and translational levels, and played crucial roles in diverse biological processes, including plant growth and development, viral defence, metabolism and apoptosis [31]. Although many progresses had been made in miRNA research in plants, including a few studies in sweet potato [32–35], the mechanisms of miRNA regulating CGA biosynthesis in leafy sweet potato remained unclear.

To better understand the basis of the high phenolic levels of leafy sweet potato, and elucidate the global expression patterns of genes and miRNAs involved in the CGA biosynthetic pathway, the present study employed transcriptome, small RNA and degradome sequencing approaches using two leafy sweet potato genotypes. These genotypes comprised one high-CGA content genotype and one low-CGA content genotype. The comprehensive and integrated analysis of different datasets identified DE mRNAs, DE miRNAs, and DE miRNA targets in CGA biosynthetic pathway, and proposed the possible CGA biosynthetic routes in leafy sweet potato.

## Material and methods

### Plant materials

The contents of TP and CGAs of which varied between the genotypes and stages, two brilliant leafy sweet potato variety and line Fushu No. 7–6 (F) and EC16 (E), which exhibited significant differences between each other from the points of genotype and stage, were chosen for further study. Variety Fushu No. 7–6 was bred by Fujian Academy of Agricultural Sciences, and introduced into Hubei Academy of Agricultural Sciences as a resource. EC16 was one of the progenies of Fushu No. 7–6. Both the genotypes were kept in the plant nursery of Food Crops Institute of Hubei Academy of Agricultural Sciences, and cultivated in potting soil on May 2^nd^, 2017, and grown outdoor potted field. The leaves of the two genotypes were sampled at two stages: 65 days (S1) and 85 days (S2) after planting. Each sample was pooled with leaves from three individual plants, and three biological replicates were collected. Part of the samples were immediately frozen in liquid nitrogen, and stored at -80°C in a freezer for transcriptomic analysis. The remaining samples were rinsed gently, and dried in a blast drier (Shangce, Wuhan, China) at 70°C. After powdered by a blender, the dehydrated samples were filtered through a 60-mesh sieve, and then were placed in the sealed plastic bags, maintaining in a freezer at -20°C for further TP and CGA measurement analyses.

### Determination of TPC

TP was determined following Xu et al [12] with a few modifications. The powders of the samples were extracted 25 times (w/v) with 70% ethanol for 40 min in an 80°C water bath. After the solution was centrifuged at 5,000 × g for 10 min, the residue was re-extracted with 70% ethanol as described above. The supernatants were combined, concentrated in a rotary evaporator, and filtered. The crude solution was diluted with distilled water to 100 ml. One ml of the prepared solution was mixed with 1 ml of Folin-Ciocalteu reagent (Guoyao, Shanghai, China), 3 ml of 7.5% Na_2_CO_3_, and 5.0 ml of distilled water in a test tube, and allowed to react at 45°C for 1.5 h in a water bath. The absorbance was measured at 756 nm using a UV-2,880 spectrophotometer (UNICO, Shanghai, China). A calibration curve of gallic acid (ranging from 0 to 0.05 mg/ml) (Guoyao, Shanghai, China) was prepared, and the TPC was expressed as mg GAE (Gallic acid equivalent) per gram of DW(Dry Weight).

### Determination of CGA contents by HPLC

The powder of the samples was extracted 50 times (w/v) with 70% ethanol for 40 min in an 80°C water bath. The solution was centrifuged at 5,000 g for 10 min, and the residue was re-extracted twice with 70% ethanol as described above. The supernatant was filtered through a cellulose acetate membrane filter (0.2 μm, Advantec, Japan), and used for analysis. A 20 μl portion of the filtrate was injected into the HPLC Agilent 1,260 system (Agilent Technologies Inc., USA) with the ZORBAX Eclipse Plus C18 column. The procedures of eluting were described as below: firstly, the column oven temperature was set at 40°C, and the mobile phase consisting of 0.1% (v/v) formic acid (A) and 100% (v/v) acetonitrile (B) was prepared. Then, the elution procedure was performed with a linear gradient as follows: 10% to 40% B from 0 to 30 min; 40% to 10% B from 30 to 30.1 min; holding at 10% B from 30.1 to 35 min. The flow rate was 0.5 ml/min, and CGAs were detected at 326 nm. The retention times of the CGA compounds were compared with standard reagents, including CQA, 3,4-CQA, 3,5-CQA and 4,5-CQA (Sigma, St. Louis, MO, USA).

### Library construction and sequencing

Total RNA was extracted from the samples using the RNeasy Plus Mini kit (Qiagen, Valencia, CA, USA). The purity, concentration and integrity of RNA were confirmed using a NanoDrop 2,000 spectrophotometer (Thermo Fisher Scientific, Wilmington, DE, USA) with an Agilent 2,100 Bioanalyzer (Agilent Technologies, Santa Clara, CA, USA). For RNA-seq, 1.5 μg RNA per sample was used as input material for rRNA removal using the Ribo-Zero rRNA Removal Kit (Epicentre, Madison, WI, USA). Sequencing libraries were generated using the NEBNext^®^ Ultra^TM^ Directional RNA Library Prep Kit following the manufacturer’s instructions. The library fragments of ~280 bp in length were purified on 2% low range ultra agrose followed by PCR amplified using phusion DNA polymerase (NEB). The products were prepared for paired-end sequencing on the Illumina High-Seq 2,500 sequencing platform (Illumina, Inc.; San Diego, CA, US). For sRNA-seq, 1 μg RNA per sample was used as input material. Sequencing libraries were generated using the NEBNext^®^ Ultra^TM^ small RNA Library Prep Kit following the manufacturer’s instructions. Shortly, cDNAs were synthesized by reverse transcription, and amplified with 12 PCR cycles. After the purification of the PCR products, deep sequencing was performed on the Illumina High-Seq 2,500 sequencing platform.

### Expression analysis and annotation

Raw data were first processed with SeqPrep (https://github.com/jstjohn/SeqPrep) and Sickle (https://github.com/najoshi/sickle) software to remove reads containing poly-N and adaptor sequences. The clean RNA-seq reads were filtered, and mapped to the *Ipomoea trilioba* (NSP323.v3) genome using HISAT (http://ccb.jhu.edu/software/hisat2/index.shtml) software [36]. The mapped reads were assembled by StringTie (https://ccb.jhu.edu/software/stringtie/). Gene expression levels were estimated using FPKM (fragments per kilobases of transcript per million fragments mapped) values calculated by RSEM (http://deweylab.biostat.wisc.edu/rsem/) software [37]. An mRNA was considered as a DE mRNA via the DESeq2 R package (http://bioconductor.org/packages/stats/bioc/DESeq2/) when it exhibited a two-fold or higher expression change, and its FDR was below 0.01 in the comparisons [38]. To functionally characterize the pathway and expression clusters, the BLAST (https://www.blast2go.com) algorithm was used to annotate DE mRNAs based on the eggNOG (http://eggnogdb.embl.de/#/app/home), KEGG (http://www.genome.jp/kegg/), Swiss-Prot (ftp://ftp.uniprot.org/pub/databases/uniprot/current_release/knowledgebase/complete/uniprot_sprot.fasta.gz), GO (http://www.geneontology.org/), Pfam (http://pfam.xfam.org/) and Nr (ftp://ftp.ncbi.nlm.nih.gov/blast/db/) databases.

For sRNAs, the clean reads were aligned to the *Ipomoea triloba* genome [39] via Bowtie2 (http://downloads.sourceforge.net/project/bowtie-bio/bowtie2/2.2.9/bowtie2-2.2.9-linux-x86_64.zip) [40], and then the reads aligned with the reference genomes were searched against miRbase (http://www.mirbase.org/) and Rfam (http://rfam.janelia.org/) to detect known miRNAs. The prediction of precursors for the novel miRNAs was performed by using miRDeep2 (https://www.mdc-berlin.de/content/mirdeep2-documentation) [41], and ones with the MFEIs of precursors (pre-miRNAs) above 0.85 were considered to be novel. Moreover, the normalized copy number of the novel miRNAs were required to be ≥10 in at least one small RNA library to avoid potential false positive. The expression levels of miRNAs in each sample were calculated, and normalized by the transcript per million (TPM) algorithm. Differential expression analyses were carried out using the DESeq R package (1.10.1). miRNAs with absolute values of log_2_ (Fold Change)≥1 and FDR≤0.05 were considered DE miRNAs.

### Degradome sequencing

Degradome library construction was conducted with the method previously described by German et al [42]. With some modifications. mRNAs were isolated by Dynabeads, and then endonucleased. The fragments containing 5’-monophosphates were ligated with 5’ adaptors, and used to generate first-strand cDNA. Single-end sequencing was performed on the Illumina High-Seq 2,500 sequencing platform, one degradome library was constructed.

### qRT-PCR

qRT-PCR analyses were carried out to determine the reliability of the RNA-seq results for expression profile analysis. All primers were designed according to the mRNA sequences and miRNA mature sequences, and were synthesized commercially in the company Tianyi Huiyuan, Wuhan. The primer sequence information was presented in S1 Table. qRT-PCR for mRNAs and miRNAs were carried out in a 20 μl system: 2.0 μl cDNA product, 10 μl 2 × qPCR Mix, and 2.5 μM for each of the forward and reverse primers. The reactions were incubated in a Real Time System Thermocycler for 10 min at 95°C, followed by 40 cycles of 15 s at 95°C, 60 s at 60°C. All reactions were run in three replicates, and β-actin served as the endogenous reference gene. The 2^-ΔΔCT^ method was employed to analyse the relative changes of genes and miRNAs [43]. T-TEST was employed to analyse the data generated from qRT-PCRs.

## Results

### Analyses of TP and CGA compositions

**Fig 2:**
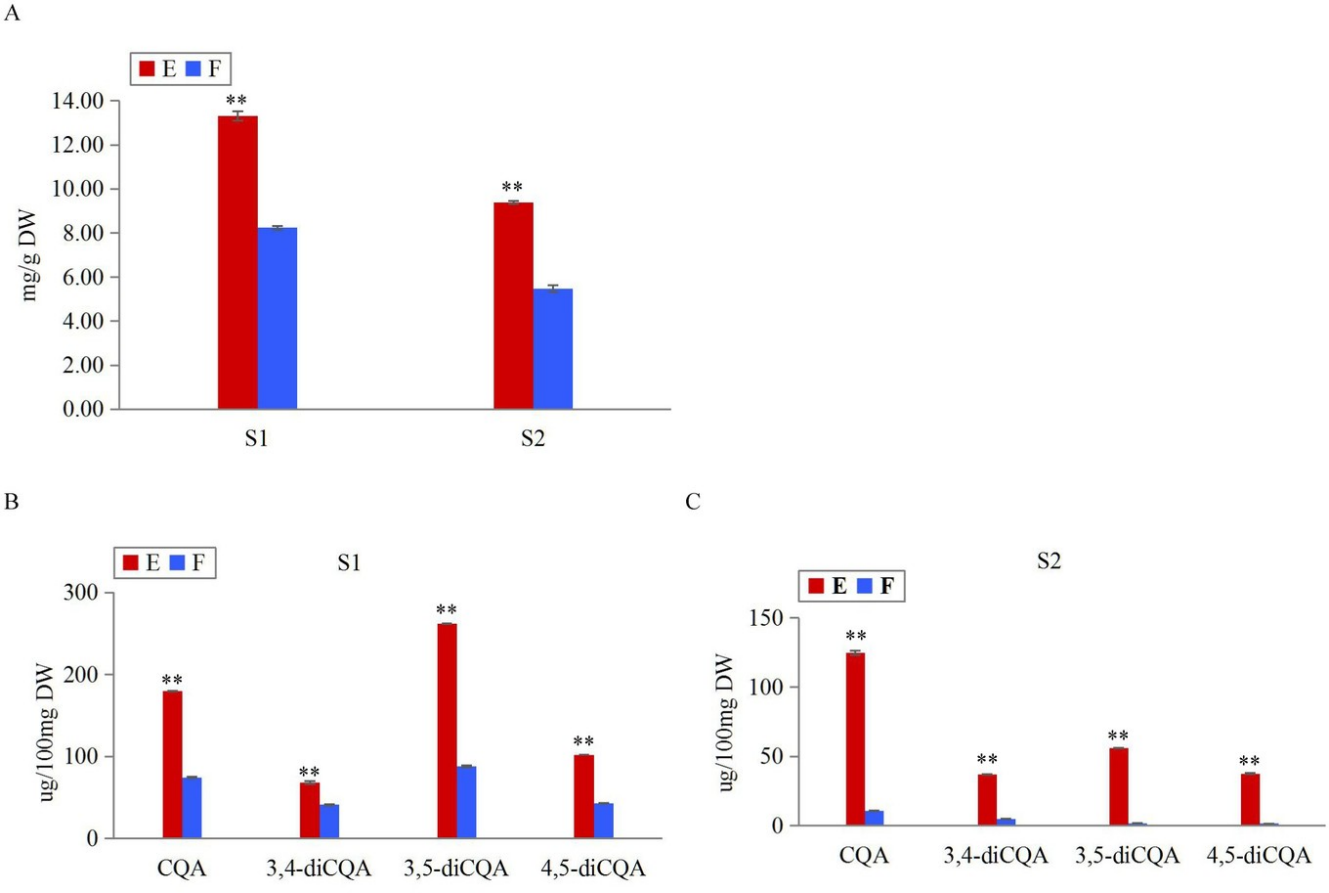
Measurements of TP and CGA contents between genotypes E and F at stages S1 and S2. (A) Measurements of TPC in genotypes E and F at stages S1 and S2. (B) Measurements of CGA monomers in genotypes E and F at S1. (C) Measurements of CGA monomers in genotypes E and F at S2. Error bars indicated ±SD (N = 3). ** T-TEST, P<0.01.

The search for CGA-related candidate genes and miRNAs was initiated by profiling two CGA-producing leafy sweet potato genotypes (E: EC16; F: Fushu No.7-6) at two stages (S1: 65 days after planting; S2: 85 days after planting) using Folin-Ciocalteu and HPLC methods. As illustrated in Fig 2A, the TPCs of E were ~1.6 and ~1.7 fold those of F at S1 and S2, respectively. In Fig 2B and 2C, at S1, the contents of CQA, 3,4-diCQA, 3,5-diCQA, and 4,5-diCQA in E were ~2.4, ~1.7, ~3.0, and ~2.4 fold those in F; at S2, the contents of CQA, 3,4-diCQA, 3,5-diCQA, and 4,5-diCQA in E were ~11.7, ~7.7, ~32.5, and ~24.7 fold those in F. Overall, the TP and CGA contents differed significantly between genotypes at each stage, and between stages of each genotype (T-TEST, P<0.01). Obviously, within the same management condition, the TP and CGA contents of E were significantly higher than F; S1 notably higher than S2.

### Transcriptome sequencing and analyses

The RNA-seq reads for two genotypes at two stages (three replicates) included 1,675.7 million reads, with individual libraries containing 128.4 to 185.7 million reads (Table 1). Reads from each sample were mapped to the reference genome using HISAT. BLAST mapping [44] revealed 29,834 (91.84%) genes with homology to protein sequences in the Nr database. The expression level distributions of expressed genes were shown in S1A Fig. Correlation analysis showed that L10 revealed low correlation to the other double replicated samples, and therefore were removed for further DE mRNA and miRNA analysis (S1B Fig).

**Table 1:** Summary of transcriptome and small RNA sequencing data.

Sample	Sample replicate	Transcriptome sequencing		Small RNA sequencing							
		No. of clean reads	Percentage of mapped reads	raw reads	No. of clean reads	17 nt < Reads <33 nt	Repeat reads	rRNA reads	tRNA reads	snRNA reads	miRNA reads
**ES1**	L01	174,683,830	76.32	25,927,605	24,095,270	24,094,597	133,724	208,381	17,766	446	106,044
	L02	166,992,924	79.98	23,865,315	22,321,800	22,320,871	141,021	395,043	14,084	684	148,547
	L03	149,408,286	79.82	32,115,499	29,998,859	29,997,913	187,042	344,301	19,841	648	211,084
**FS2**	L04	138,978,770	80.06	21,378,200	20,333,242	20,330,797	92,533	905,305	97,290	2,787	286,047
	L05	141,222,622	84.71	23,317,399	22,270,590	22,266,892	102,186	486,652	50,227	1,061	499,324
	L06	139,989,810	81.51	15,117,444	14,387,309	14,385,108	58,805	292,689	49,024	1,352	285,295
**ES2**	L07	153,472,418	80.64	26,286,171	24,518,825	24,518,144	159,900	1,034,571	50,200	1,601	171,786
	L08	142,547,416	76.26	29,131,977	27,250,685	27,248,979	148,601	296,936	36,413	631	365,179
	L09	128,383,618	78.67	18,473,381	17,240,894	17,240,236	102,292	209,377	32,750	541	201,224
**FS1**	L11	154,290,016	80.96	24,627,110	23,088,334	23,087,243	113,446	296,534	17,196	470	227,489
	L12	185,704,656	79.65	24,673,614	23,078,309	23,077,146	113,972	316,806	12,650	565	211,848
**Total**		1,675,674,366		264,913,715	248,584,117	248,567,926	1,353,522	4,786,595	397,441	10,786	2,713,867

Notes: E denoted EC16; F denoted Fushu No. 7–6; S1 denoted 65 days after planting; S2 denoted 85 days after planting. L01-L12 denoted different sample.

### Differentially expressed gene analyses and annotation

**Fig 3:**
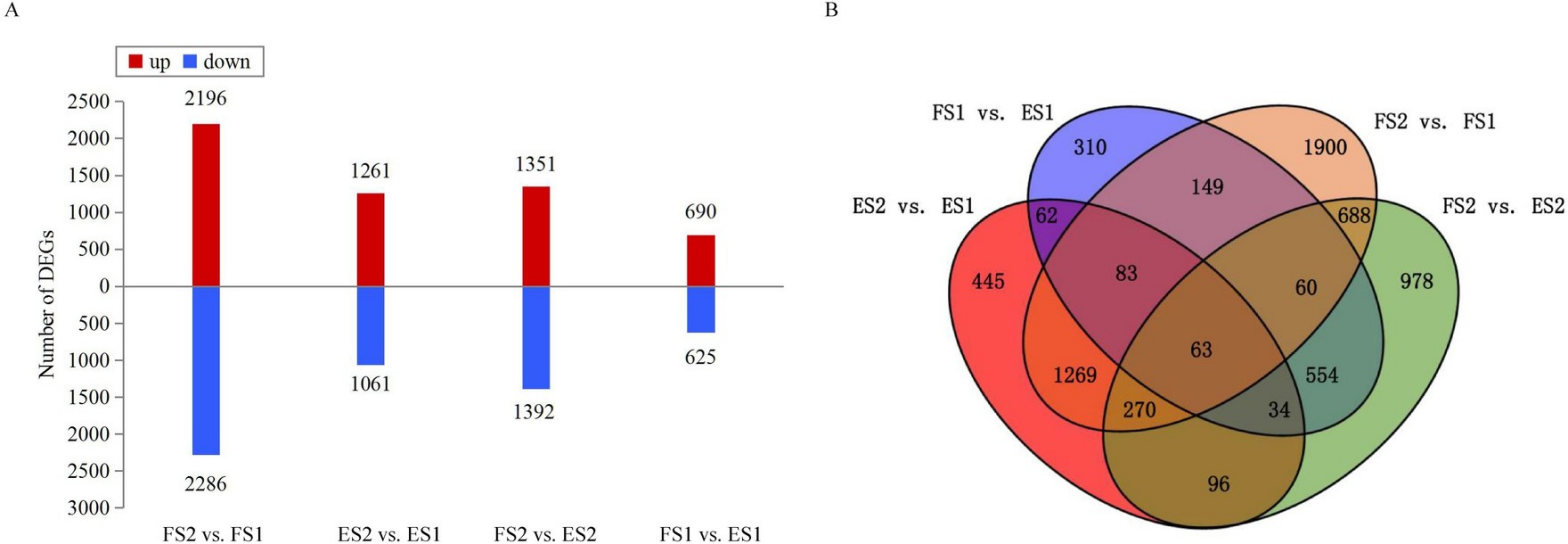
DE mRNA analyses. (A) Numbers and levels of DEGs in the four comparisons. (B) Venn diagram of DE mRNAs among the four comparisons.

The TP and CGA compounds increased across the four pairwise comparisons (FS2 vs. FS1; ES2 vs. ES1; FS2 vs. ES2; FS1 vs. ES1), thus co-regulated DEGs across the comparisons indicated the pivotal steps in the pathway of CGA biosynthesis. In total, 6,961 DEGs were found across the four comparisons (S2 Table). The number of DEGs ranged from 1,315 (690 upregulated; 625 downregulated) for FS1 vs. ES1 to 4,482 (2,196 upregulated; 2,286 downregulated) for FS2 vs. FS1 (Fig 3A). A total of 1,685 and 711 DEGs exhibited common expression patterns between FS2 vs. FS1 and ES2 vs. ES1, between FS1 vs. ES1 and FS2 vs. ES2; an overlap of 63 common DEGs were found across the four comparisons (Fig 3B). In comparisons of stage-specific and genotype-specific groups, 2,333 common DEGs that were identified at least in two comparisons were then considered for further analysis.

**Fig 4:**
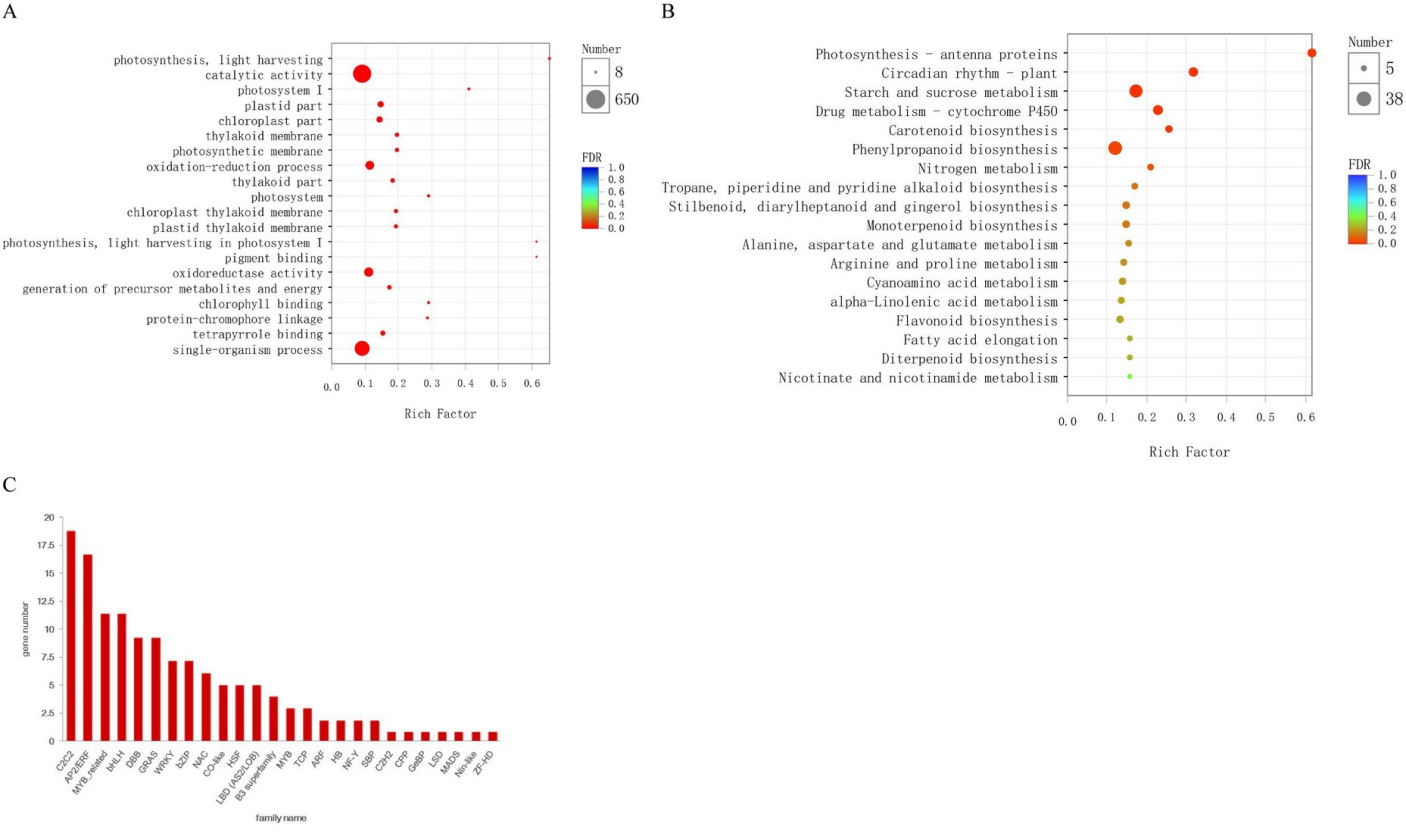
Annotation and pathway analyses of the identified DEGs. (A) GO enrichment analysis of common DEGs. (B) The scatter plot of enriched KEGG pathways of DEGs. (C) Number of genes from TF families in the common DEGs.

To functionally characterize expression genes, firstly, we used the BLAST algorithm to annotate 6,961 DEGs based on the eggNOG, KEGG, Pfam, GO, Nr and Swiss-Prot databases. As a result, 4,426, 2,289, 2,678, 1,273 DEGs were annotated for FS2 vs. FS1, ES2 vs. ES1, FS2 vs. ES2, FS1 vs. ES1; detailed annotation information was provided in S3 Table. Out of these DEGs, 2,333 common DEGs were assigned to 47 GO terms in S4 Table, including the biological process (20), molecular function (14) and cellular component (13) categories. The GO enrichment analysis of common DEGs revealed that catalytic activity (GO:0003824), oxidation-reduction process (GO:0055114), oxidoreductase activity (GO:0016491) ranked in the top 20 most significant enriched terms in Fig 4A. Furthermore, the pathway analysis of common DEGs was carried out to understand the molecular mechanism using the KEGG database. The DEGs were found to represent 288 pathways (S5 Table). The enrichment analysis suggested that photosynthesis-antenna proteins (map00196), starch and sucrose metabolism (map00500), drug metabolism-cytochrome P450 (map00982) and phenylpropanoid biosynthesis (map00940) were among the most enriched pathways (Fig 4B). A total of 134 transcription factors (TFs) belonging to 26 families were identified differentially expressed. Among them, *C2C2* (18), *AP2/ERF* (16), *MYB-related* (11) and *bHLH* (11) were the most overrepresented TF families (Fig 4C).

### Metabolic pathway and gene analyses during CGA accumulation

**Fig 5:**
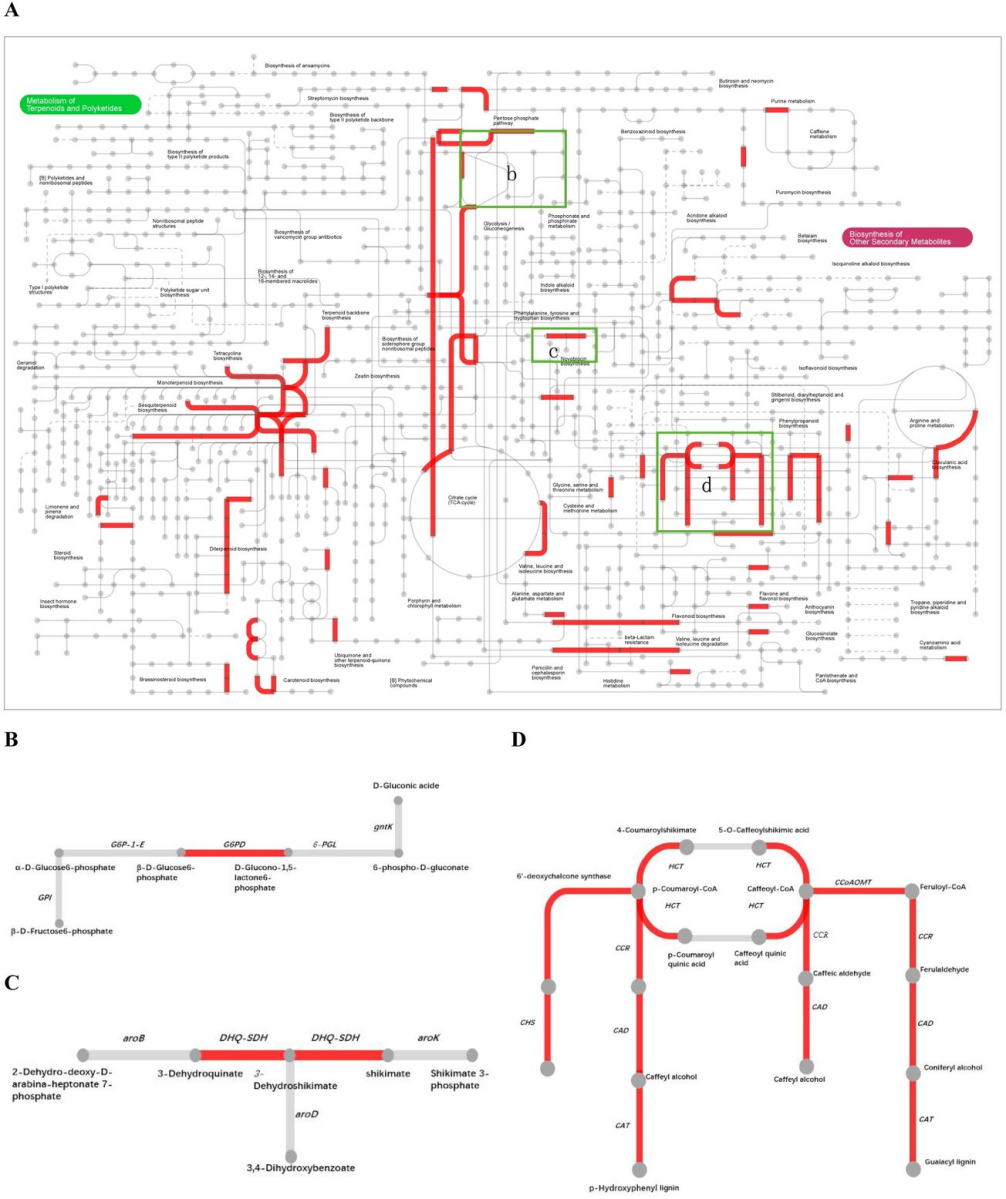
Interactive pathways analyses during CGA accumulation. (A) The red lines indicated genes with significant expression change. (B) Pentose phosphate metabolism. (C) Phenylalanine biosynthesis. (D) CGA biosynthesis.

To provide a global view of leafy sweet potato secondary metabolism, common DEGs with different map ids were further submitted for analysis via the online Interactive Pathway (ipath) explorer v2 (https://pathways.embl.de/) (Fig 5A) [45]. The metabolic pathways such as pentose phosphate pathway (Fig 5B), phenylalanine biosynthetic pathway (Fig 5C), CGA biosynthetic pathway (Fig 5D) and flavonoid biosynthesis showed enhanced, which were in accordance with the results of GO analysis. As pentose phosphate metabolism, phenylalanine biosynthetic pathway and CGA biosynthetic pathway were vital steps for CGA biosynthesis, genes involving in these pathways were fully illustrated.

**Fig 6:**
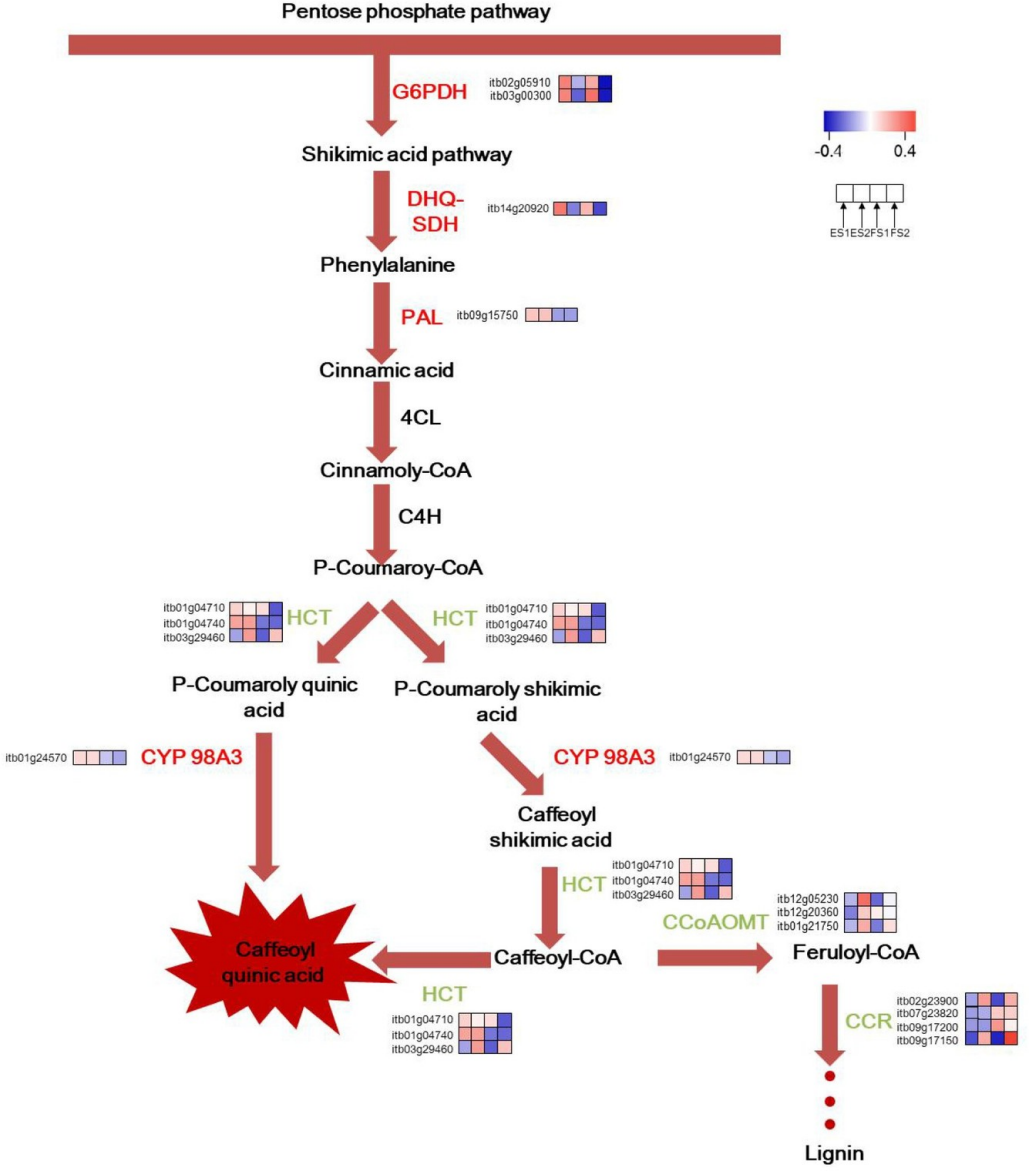
A hypothetical representation of the expression patterns of genes involved in the CGA biosynthetic pathway in leafy sweet potato. Upregulated Genes were shown in red, downregulated genes in green and non-DEGs in black.

In this work, genes participating in CGA biosynthesis showed special expression patterns. For the common expression pattern, two synonymous DEGs of *G6PDH* (itb02g05910; itb03g00300) which encoded glucose-6-phosphate 1-dehydrogenase were upregulated, providing sufficient NADPH and erythrose 4-phosphate(E4P) for shikimic acid pathway. *DHQ*/*SDH* (itb14g20920) which catalyzed the biosynthesis of phenylalanine was upregulated as well. *HCT* that had three synonymous DEGs (itb03g29460; itb01g04710; itb01g04740) expressed differentially across the four comparisons. Yet itb03g29460 was the most expressed one, which was downregulated in ES2 vs. ES1 and FS2 vs. FS1. *CCR*, which encoded cinnamoyl-CoA reductase, had 4 synonymous DEGs (itb09g17150; itb09g17200; itb02g23900; itb07g23820). They were downregulated in different comparisons. The *CCoAOMT* that encoded caffeoyl-CoA O-methyltransferase had three synonymous DEGs (itb12g05230; itb01g21750; itb12g20360), all of which were downregulated. In this study, *PAL* (itb09g15750) was found to be only upregulated in the comparison of FS2 vs. ES2. The *C3’H* (p-coumarate 3′-hydroxylase), another name *CYP 98A3* (itb01g24570) was upregulated in the comparison FS2 vs. ES2. The schematic of metabolic data related to leafy sweet potato CGA accumulation was briefly illustrated in Fig 6.

### High-throughput small RNA sequencing

**Fig 7:**
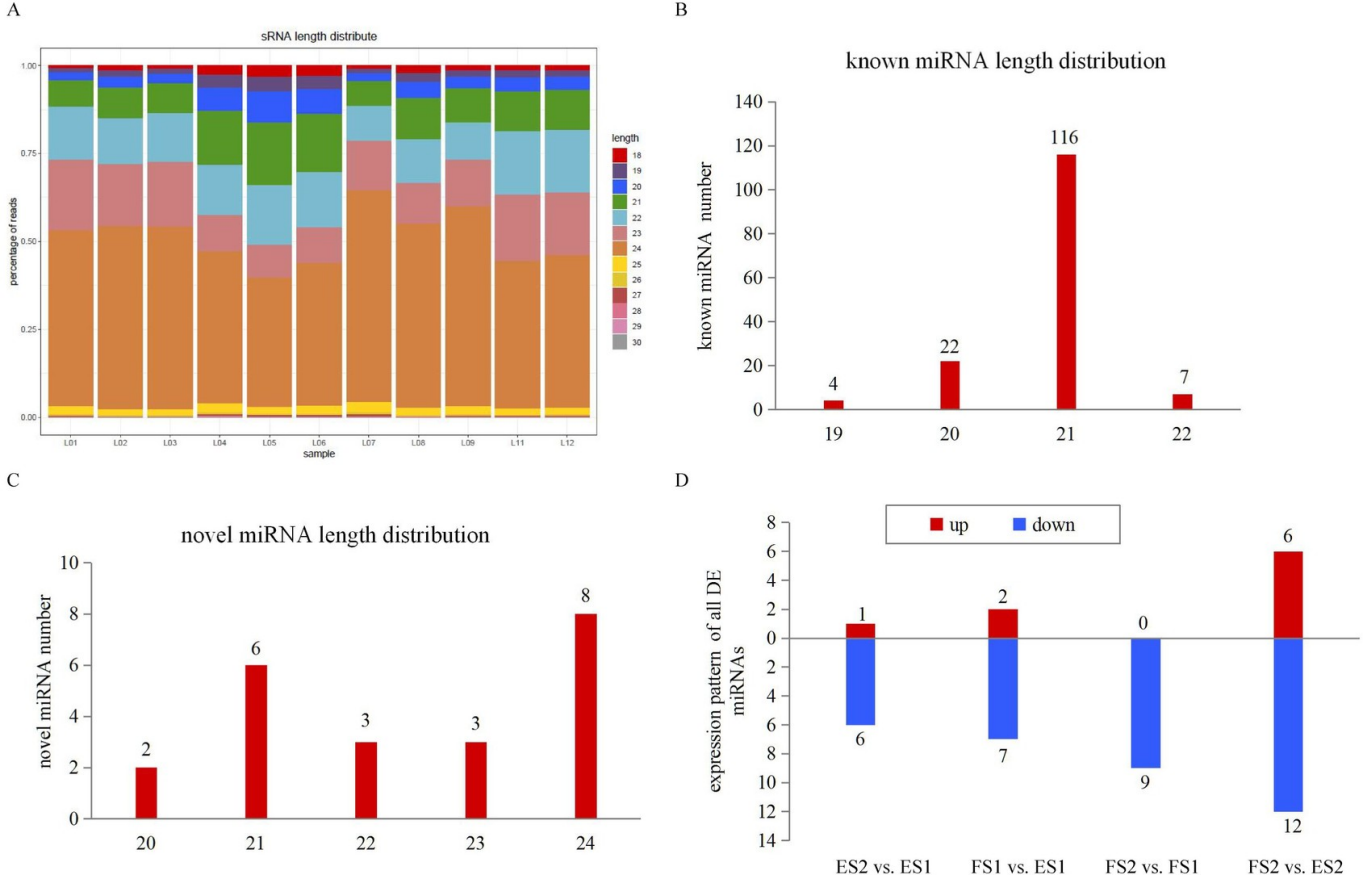
Size distribution of miRNAs by sRNA-seq. (A) Length distribution of sRNAs. (B) Numbers of length distribution of known miRNAs. (C) Numbers of length distribution of novel miRNAs. (D) Expression pattern of DE miRNAs.

The small RNA sequencing resulted in 248.6 million clean reads, with 14.4 to 30.0 million reads per library. Reads with length > 17 nt and < 33 nt were kept, following by the removal of ribosomal RNA (rRNA), transfer RNA (tRNA), small nucleolar RNA (snoRNA), and repetitive sequences (Table 1). The length distribution patterns of the sRNAs were similar in the eleven libraries. They ranged from 18 to 30 nt, of which 24 nt were the most abundant size (Fig 7A), similar to the results reported in previous research in sweet potato and other species [35,46]. In order to identify known miRNAs, the filtered reads were searched against the miRNAs in miRBase. A total of 149 known miRNAs were obtained, the most length distribution was 21 nt (Fig 7B). As some of the known miRNAs were aligned with more than one pre-miRNAs, the detailed information of all aligned miRNAs was listed in S6 Table. Reads that could not be mapped to miRBase were subjected to novel miRNA predication by miRDeep2, and the most length distribution of novel miRNA was 24 nt following by 21 nt (Fig 7C). A total of 22 novel miRNAs were identified, and listed in S6 Table. The negative folding free energies of the hairpin structures of novel miRNAs ranged from -68.37 to -26.52 kcal mol^-1^ with an average of -43.47 kcal mol^-1^. The minimal folding free energy index (MFEI) of novel miRNAs ranged from 0.86 to 1.69 with an average of 1.13.

### DE miRNA expression during CGA accumulation

miRNAs were considered as DE miRNAs if they had absolute values of log_2_ (Fold Change) ≥1 and FDR (False discovery rate) ≤0.05. A total of 9, 7, 18 and 9 miRNAs were identified as DE miRNAs across the four comparisons, and 5 miRNAs were common differently expressed ones (S7 Table). The majority of DE miRNAs showed a trend of down-regulation during CGA accumulation (Fig 7D). miR156, miR166, miR167 and miR858 were found in different comparisons, which had been reported to be involved in phenylpropanoid pathway [47].

### Target predication via in silico and degradome approaches

**Fig 8:**
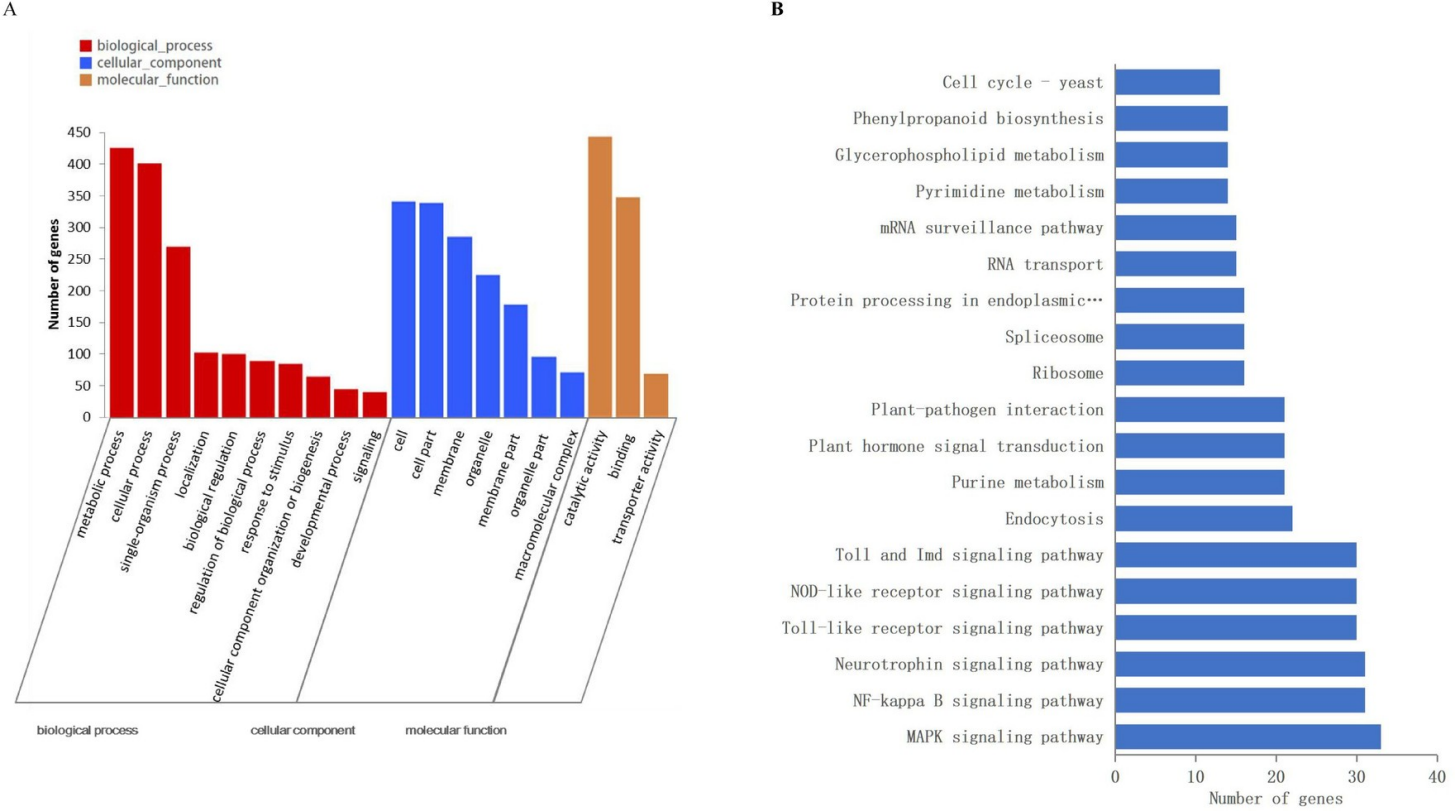
DE miRNA target genes’ annotations. (A) GO analysis of annotated DE miRNA target genes. (B) Major pathways of all annotated DE miRNA target gene.

To explore the function of miRNAs, computational program was performed to predict their target genes. All identified 171 miRNAs were predicated to have 1,799 targets via TargetFinder software with the score value < 4 [48]. The annotations for the 1799 miRNA targets were based on the GO, KEGG, eggNOG, Nr, Swiss-Prot and Pfam databases (S8 Table). The targets were uniformly assigned to 20 biological processes, 14 cellular components and 11 molecular functions. The most abundant 20 GO terms were demonstrated in Fig 8A. The significant enriched GO terms like lignin catabolic process (GO:0046274), phenylpropanoid catabolic process (GO:0046271), lignin metabolic process (GO:0009808) and phenylpropanoid metabolic process (GO:0009698) were listed in S9 Table, and they were all involved in CGA accumulation pathway. Furthermore, KEGG annotation was carried out to explore the pathways in which the identified targets were involved. A total of 220 pathways were identified indicating the highly diverse functions of the targets. Phenylpropanoid biosynthesis (map00940) which was CGA accumulation-related pathway were among the most 20 abundant pathways (Fig 8B).

Using degradome sequencing, a total of 21.94 Mb clean tags and 7,892,630 cluster tags were obtained. The cluster tags were aligned to the transcriptome and Rfam database for cleavage site analysis. After processing and analysis with CleaveLand software [49], 158 miRNA-mRNA pairs were totally identified (S10 Table). Target analysis showed that many cleaved-target genes by miRNAs were TF genes, including *AP2/ERF*, *bZIP*, *TCP*, *MYB*, *SPL*, etc. Some miRNAs had more than one target genes, like miR530a targeted *microtubule-associated protein 70-1-like* and *bHLH130-like* genes. On the contrary, the same gene can be targeted by more than one miRNA, for instance, miR394c and miR384-5p shared the same target *F-box*. TFs such as *AP2/ERF* (itb14g16290) and *SPL* (itb01g24030) predicated *in silico* were validated in degradome sequencing.

### Correlation analysis of DE miRNA expression profiles and their target genes

The expression of both DE miRNAs (from small RNA-seq) and their target genes (from RNA-seq) were integrated to infer the mediatory role of miRNAs during CGA biosynthesis. Coherent interactions were the ones in which the expression of miRNA was upregulated when the expression of target mRNA was downregulated, and vice versa. In this study, the spearman mathematical method with the criteria of index < = -0.8 and P-value < = 0.05 was employed. As a result, the correlation analysis of DE miRNA and their target mRNA expression profiles identified a total of 205, 8, 40 and 1 miRNA-mRNA interaction pairs across four comparisons (FS2 vs. FS1; ES2 vs. ES1; FS2 vs. ES2; FS1 vs. ES1) (S11 Table). From these coherent pairs, stu-miR156g-5p, stu-miR156k-5p, stu-miR156i-5p, stu-miR156h-5p, stu-miR156j-5p, stu-miR156e were found downregulated, their target gene AP2/ERF (itb11g02720) and *SPL* (itb02g07930) were upregulated in FS2 vs. FS1, the negative correlation of which was -0.9; ath-miR858a and ath-miR858b was upregulated, and its target *MYB* (itb12g01510) was downregulated in FS2 vs. ES2, the negative correlation of which was -0.8.

#### Validation of differential gene and miRNA expression

**Fig 9:**
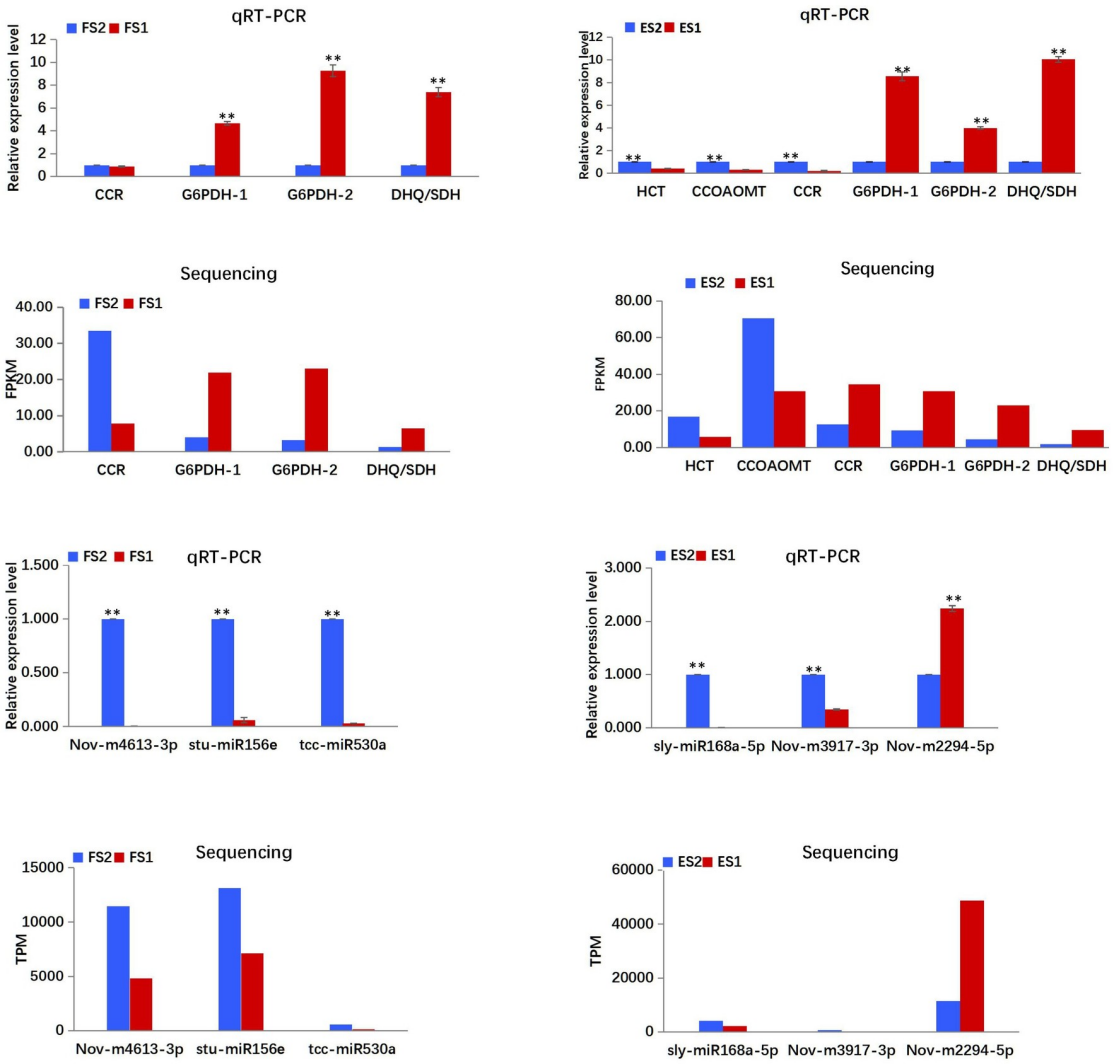
Expression patterns of mRNAs and miRNAs determined by qRT-PCR. Expression levels of mRNAs and miRNAs were normalized according to the level of β-actin in qRT-PCRs. Three biological replicates were performed for each mRNA and miRNA. T-TEST was employed to analyse the data. The data were presented as the mean ± SD. **P<0.01.

The qRT-PCR analysis was carried out to validate the expression patterns of genes and miRNAs obtained from the RNA and small RNA sequencing. The expression of enzyme encoding-genes (*HCT*, *CCoAOMT*, *CCR*) in the CGA biosynthetic pathway, two synonymous *G6PDH* genes (itb03g00300, itb02g05910) in the pentose pathway, and one phenylalanine biosynthesis-related gene *DHQ/SDH* were validated via qRT-PCR (Fig 9), and the results were consistent with that of the mRNA sequencing, except the deviations of *CCR* in ES2 vs. ES1. In addition, six miRNAs, namely, Nov-m2294-5p, Nov-m3917-3p, Nov-m4613-3p, sly-miR168a-5p, stu-miR156e and tcc-miR530a were validated by qRT-PCR as well (Fig 9; S1 Table). Similar expression trends (upregulated or downregulated) were observed between the qRT-PCR analysis and the sRNA sequencing result.

## Discussion

Leafy sweet potato is extremely popular among consumers in China, because it is beneficial to health. CGAs in leafy sweet potato are not only the key attributes for health care by fresh consuming, but also have potential applications in food and pharmaceutical industries. As a result, the major objective of this research is to comprehensively study the CGA metabolism, and investigate the molecular basis of this pathway in leafy sweet potato. The availability of diverse germplasm resources and high-throughput approaches, namely, transcriptome, small RNA and degradome sequencing, provide an opportunity to dissect the mechanism.

We investigated two well-characterized genotypes at two stages for their different CGA accumulation (Fig 2A–2C). Although a few genes involved in CGA biosynthesis had been reported in other plant species, such as tomato [21,22], *Arabidopsis* [23,24], *Nicotiana tabacum*[27], *Lonicera japonica* [50,51], Sunflower [52], Dandelions [53,54], *Cecropia obtusifolia* [55], the molecular mechanisms underlying in leafy sweet potato remains largely unknown. In the present study, a total of 29834 genes were identified based on the reference guided transcriptome analysis of two genotypes at two stages. The sequences for all genes in route 2 and 3 were assembled, and the differentially expressed functional genes were concentrated in these two routes. Therefore, it was reasonable to conclude that route 2 and 3 were the main pathways for the biosynthesis of CGA in leafy sweet potato. This result was not in accordance with that of the report by Kojima and Uritani [19], in which the biosynthesis of CGA was assumed by route 1. We speculated that the mechanism of CGA biosynthesis in the leaves of sweet potato was different from that of the root.It has been acknowledged that the accumulation of CGAs is a multifaceted process that can be traced to the pentose phosphate pathway, where the precursor E4P is produced. Following this process is the shikimic acid pathway, which is the main pathway leading to the production of phenylalanine, and then CGAs are produced by the catalysis of enzyme cascades [18,56,57]. The formations of E4P, biosynthesis of phenylalanine and CGAs were supported by the KEGG enrichment analysis presented in the study. The expression of the important enzymes of *G6PDH* and *DHQ/SDH*, which involved in the biosynthesis of E4P and phenylalanine, were integrally upregulated in high-CGA content samples compared to low-CGA content samples. In this study, ipath that integrated 123 KEGG maps from 183 species was employed as an open access online tool to indicate the enhanced catalytic activities. From the ipath map, pathways of pentose phosphate, phenylalanine biosynthesis, phenylpropanoid biosynthesis and flavonoid biosynthesis were all indicated in red, meaning related genes were active in these pathways.

As two possible routes (route 2 and 3) for CGA biosynthesis were indicated in the research, the initiation study for both routes started from DEG *PAL*. Phenylalanine was catalyzed by PAL to form cinnamic acid. DEG *PAL* in this study was found only in FS2 vs. ES2 instead of the common DEG aggregation. In addition, the following downstream two enzyme genes, *4CL* and *Cinnamate 4-hydroxylase* (*C4H*), which catalyzed the formation of p-Coumaroy-CoA, did not express significantly differently among comparisons in this research. These results were quite different from other higher plants, for them *PAL*, *4CL* and *C4H* were key enzymes in CGA accumulation [58]. However, the intriguing phenomenon occurred on three common DEGs *HCT*, *CCR* and *CCoMAT* in the phenylalanine pathway. The expression of *CCR* and *CCoMAT* were downregulated indicating the lignin biosynthesis was altered to CGA accumulation. For *HCT*, all homologous genes were upregulated except the most highly expressed one (itb03g29460). Though having been shown to synthesize caffeoylquinate *in vitro* [16], *HCT* was involved both upstream and downstream of the 3-hydroxylation step (Fig 1). Its inhibition could affect predominant caffeoylquinate catabolizing into caffeoyl CoA which led to the lignin biosynthesis, and thus the CGA accumulation occurred. The same phenomenon had been reported by Hoffmann et al [17]. The CGA mechanism also involved a number of TFs like *C2C2*, *AP2/ERF*, *MYB-related*, *bHLH*, etc.

miRNAs have emerged as master modulators of gene expression and are promising tools for crop improvement [59]. A few studies in sweet potato had reported the genome-wide discoveries of miRNAs [60–62], but no study had yet characterized the roles of miRNAs in CGA biosynthesis. In the present study, a total of 149 known and 22 novel miRNAs were identified. The expression pattern of the isolated miRNAs were analyzed, more miRNAs were downregulated than upregulated across the four comparisons (Fig 7D), and thirty-eight miRNAs were recognized as DE miRNAs. Most of the DE miRNAs were known miRNAs. miR156, miR166, miR167 and miR858 family members were confirmed, which were reported to be negatively involved in regulating catechin and anthocyanin biosynthesis [63–65]. As catechin and anthocyanin belonged to flavonoid pathway which had close correlations with CGAs, stu-stu-miR166d-3p, stu-miR166c-3p, stu-miR166a-3p, stu-miR166b, sly-miR167b-3p, stu-miR156a, stu-miR156b, stu-miR156c, stu-miR156d-5p, stu-miR156j-5p, stu-miR156k-5p, stu-miR156g-5p, stu-miR156h-5p, stu-miR156i-5p, stu-miR156e, ath-miR858a, ath-miR858b identified in this study were potential targets for manipulation CGA contents for further study.

Degradome sequencing that had been successfully applied to identify miRNA targets in many plant species [66,67] were employed to verify the predication results. In degradome sequencing analysis, the majority of target genes were transcription factors, containing *SPL*, *HD-ZIP* and *MYB* genes. These TFs were all reported to be related to the phenylpropanoid pathway. For instance, *SPLs* played important roles in plant growth and development. The miR156/*SPL* module was reported to participate in the biosynthesis of phenylpropanoids by destabilizing the MYB-bHLH-WD (MBW) complex, and directly preventing the expression of anthocyanin biosynthetic genes in *Litchi chinensis* [68]; as *HD-ZIP* TFs played crucial roles in shoot apical meristem and organ polarity, the blockage of miRNA165/166 caused the upregulation of *HD-ZIP* TFs, and increased IAA content accompanied by enhanced anthocyanin [58]. In this research, the analyses of degradome sequencing demonstrated that *SPLs* were targeted by miR156, *HD-ZIP* by miR166, *MYB* by miR159. These results suggested that miR156, miR166 and miR159 might be involved in CGA biosynthetic pathway.

The miRNA and target mRNAs association analysis revealed that miR156 and miR858 families were strongly negatively correlated with the mRNAs. miR156g-5p, miR156k-5p, miR156i-5p, miR156h-5p, miR156j-5p, miR156e in FS2 vs. FS1 commonly negatively targeted DEG AP2/ERF (itb11g02720) and *SPL* (itb02g07930); ath-miR858a and ath-miR858b commonly negatively targeted *MYB* (itb12g01510). All these target genes were noted to be associated with CGA biosynthesis.

## Conclusions

In summary, the present study integrated mRNA and miRNA expression data along with degradome analysis to identify key factors in CGA biosynthesis in leafy sweet potato. The study revealed complex mechanism, in which pentose metabolism and lignin biosynthesis were all related to CGA biosynthesis, and routes 2 and 3 were the possible CGA biosynthetic pathway in leafy sweet potato. A set of genes and miRNAs were identified as crucial roles for the CGA biosynthesis. They could serve as targets for further research of gene functions. This study provided a foundation for uncovering the CGA biosynthetic system in leafy sweet potato, and the results could be used to improve leafy sweet potato varieties for both consumer health benefits and pharmaceutical use in the future.

## Supporting information

S1 Table Sequence information of mRNAs and miRNAs for qRT-PCR analyses. (XLSX)

S2 Table DEGs identified across the four comparisons. (XLSX)

S3 Table Detailed annotation information of the four comparisons. (XLSX)

S4 Table GO term analysis of DEGs. (XLSX)

S5 Table KEGG analysis of DEGs. (XLSX)

S6 Table Known and novel miRNAs identified across the four comparisons. (XLSX)

S7 Table DE miRNA expression patterns. (XLSX)

S8 Table The annotation of identified miRNAs targets. (XLSX)

S9 Table GO enrichment analysis of the identified miRNAs targets. (XLSX)

S10 Table miRNA targets identified from degradome sequencing. (XLSX)

S11 Table Association analysis between DE miRNAs and mRNAs. (XLSX)

S1 Fig Results of RNA-seq transcriptome assembly. A Numbers and levels of expressed genes from different samples. B Correlation-based clustering analyses of RNA-seq expression across all replication.
(PDF)
