# Supplementary figures and images for: Integrated transcriptome, small RNA and degradome sequencing approaches proffer insights into chlorogenic acid biosynthesis in leafy sweet potato
Source: PLoS One. 2021 Jan 22;16(1):e0245266. doi: 10.1371/journal.pone.0245266 (PMC7822329; doi:10.1371/journal.pone.0245266)

A

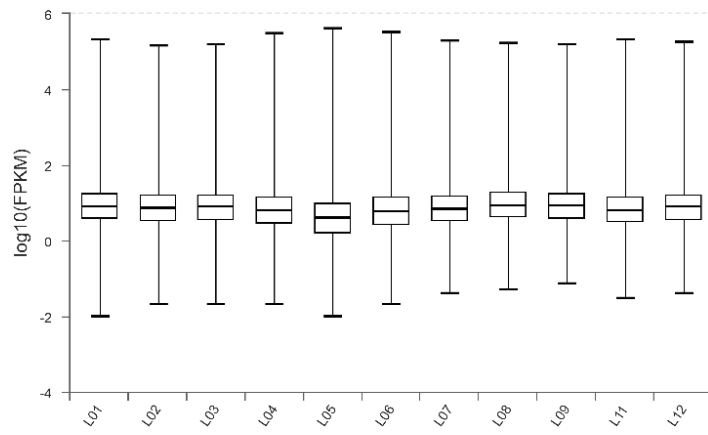**B**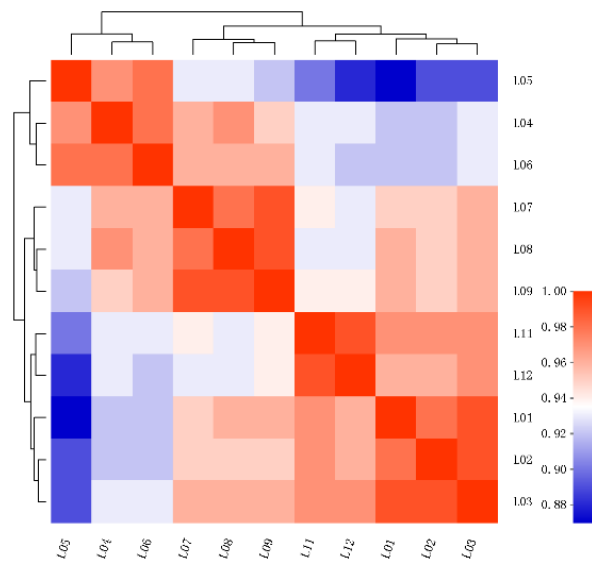

Supplement: S1 Fig — A Numbers and levels of expressed genes from different samples. B Correlation-based clustering analyses of RNA-seq expression across all replication. (PDF) [file pone.0245266.s012.pdf]
